# Reduced Expression of m^6^A Demethylases *FTO* and *ALKBH5* in Monocytes from the Site of Inflammation in Patients with Juvenile Idiopathic Arthritis

**DOI:** 10.3390/ijms26189248

**Published:** 2025-09-22

**Authors:** Hisham I. Abu-Tawil, Lucas W. Picavet, Ellen C. N. van Vroonhoven, Alejandra Bodelón, Rianne C. Scholman, Nienke ter Haar, Arjan Boltjes, Sebastiaan J. Vastert, Jorg van Loosdregt

**Affiliations:** 1 Center for Translational Immunology, University Medical Center Utrecht, 3508 GA Utrecht, The Netherlands; h.i.m.abutawil@umcutrecht.nl (H.I.A.-T.); luuk.pica@gmail.com (L.W.P.); a.bodelondefrutos@umcutrecht.nl (A.B.); r.c.scholman@umcutrecht.nl (R.C.S.); b.vastert@umcutrecht.nl (S.J.V.); 2 Department of Laboratory and Blood Bank, King Faisal Medical City for Southern Regions, Ministry of Health, Abha 62523, Saudi Arabia; 3 Department of Pediatric Rheumatology and Immunology, University Medical Center Utrecht, 3508 GA Utrecht, The Netherlands; nmterhaar@gmail.com

**Keywords:** m^6^A RNA methylation, FTO, ALKBH5, monocytes, juvenile idiopathic arthritis, TNFα, autoimmune disease

## Abstract

N^6^-methyladenosine (m^6^A) has recently emerged as a post-transcriptional modulator governing cell-specific gene expression in innate immune cells, particularly in monocytes. Disruptions in m^6^A homeostasis, manifested as the altered expression of m^6^A-related proteins and m^6^A levels, have been implicated in autoimmune disorders. Perturbations in m^6^A dynamics within total Peripheral blood mononuclear cells (PBMCs) have shown strong correlations with disease severity in rheumatoid arthritis (RA) and systemic lupus erythematosus (SLE). It remains unclear in which specific cell type(s) m^6^A homeostasis is disturbed, and also whether other rheumatic diseases such as juvenile idiopathic arthritis (JIA) show similar features. Here, we assess the involvement of m^6^A and m^6^A-regulatory proteins in JIA monocytes. Notably, the diminished expression of m^6^A-eraser proteins FTO and ALKBH5 was observed in JIA monocytes extracted from the inflamed joint. This resulted in increased m^6^A-methylated transcripts in monocytes from these patients. Correspondingly, we observed that culturing monocytes in the presence of synovial fluid from JIA inflamed joints reduced the expression of both *FTO* and *ALKBH5*. The knock-out of *FTO* in human monocytes of healthy controls increased monocyte activation, indicating the relevance of *FTO* and m^6^A in the context of JIA. These findings underscore the potential of *ALKBH5* and *FTO* expression as a biomarker in JIA and identify the m^6^A machinery as a potential therapeutic target for the treatment of JIA and possibly other autoimmune diseases in the future.

## 1. Introduction

Autoimmune disorders exhibit a complex immune pathogenesis involving multiple factors, prominently influenced by genetic and environmental elements triggering auto-immune pathways ultimately leading to chronic immune activation and tissue damage [1,2,3,4]. These disorders are principally characterized by the response of the adaptive immune system against self-components and the presence of auto-antibodies. Nevertheless, the activation of T and B cells, which can identify the self-antigens, requires stimulation by the innate immune system. Innate immune cells, including monocytes and neutrophils, play a vital role in producing diverse cytokines and chemokines which attract immune cells to inflammation sites and thereby fuel the inflammation in various autoimmune diseases [5]. In accordance with this, monocyte infiltration has been documented at the site of inflammation in both rheumatoid arthritis (RA) and juvenile idiopathic arthritis (JIA) [6,7].

JIA describes a range of chronic arthritic conditions that first manifest in individuals before the age of 16 and persist for at least six weeks, with no identifiable underlying cause. The disease is characterized by joint inflammation and can have various clinical presentations and outcomes. Moreover, elevated pro-inflammatory cytokines produced by monocytes, such as TNF, have been implicated in JIA pathology [8,9,10]. However, the fundamental mechanisms behind the increased activation of the innate immune system remain unclear.

Disturbed epigenetic regulation in monocytes has been demonstrated to contribute to the pathogenesis of autoimmune diseases including JIA [11,12,13,14]. More recently, the post-transcriptional modification of N^6^-methyladenosine (m^6^A) has been implicated in the regulation of gene expression in various immune cell types. m^6^A is the most prevalent mRNA modification, regulated by different methylases and demethylases (‘writer’ and ‘eraser’ proteins), rendering it a dynamic modification responsive to internal cellular cues and environmental signals. Previously, m^6^A has been shown to be pivotal for macrophage polarization, inflammatory responses and monocyte activation [15,16,17,18,19,20,21].

m^6^A and its regulatory proteins are implicated in autoimmune disease pathogenesis. In the context of rheumatoid arthritis (RA), increased expression of m^6^A writer proteins and reduced expression of m^6^A eraser proteins fat mass and obesity -associated protein (FTO) and AlkB homolog 5 (ALKBH5) have been reported to correlate with disease severity [22]. Moreover, an increased expression of methyl transferase-like 3 (METTL3, catalytic core component of the m^6^A writer complex) has been observed in RA PBMCs, and its expression correlates with C-reactive protein (CRP) and erythrocyte sedimentation rate (ESR) levels in serum [18]. Additionally, GWAS studies have identified 37 SNPs in RA that could potentially influence the m^6^A methylation of RNA as they are located in critical flanking nucleotides of predicted m^6^A sites [23]. In various autoimmune disorders, m^6^A has been implicated in disease pathogenesis, including systemic lupus erythematosus (SLE) [24,25,26,27], psoriasis [28], and multiple sclerosis (MS) [29].

Although the role of m^6^A in JIA remains poorly understood, m^6^A regulators and modification patterns have been identified as potential diagnostic biomarkers for JIA [30]. Here, reduced FTO expression has been associated with immunological activation and increased activated dendritic cell counts in patients [30]. Additionally, decreased FTO expression in PBMCs was correlated with a higher expression of co-stimulatory molecules, adhesion molecules, and MHC molecules, suggesting that FTO and m^6^A might contribute to dendritic cell activation and hyperactive immune responses in JIA patients.

In this study, we investigated the role of m^6^A and its regulating proteins in monocytes from JIA patients. We demonstrate that JIA monocytes derived from inflamed joints display reduced *ALKBH5* and *FTO* expression and increased m^6^A levels. Correspondingly, the exposure of healthy control monocytes to synovial fluid from inflamed JIA joints decreased both *ALKBH5* and *FTO* expression in these cells, indicating that the expressions of *ALKBH5* and *FTO* are regulated by components in the inflamed synovial environment. Furthermore, we demonstrate that reduced *FTO* expression promotes monocyte activation in vitro, evident by increased TNF expression. Collectively, these data underscore the role of m^6^A in JIA pathogenesis, and suggest that *ALKBH5* and *FTO* could serve as a potential biomarker in JIA.

## 2. Results

### 2.1. JIA Synovial Monocytes Exhibit Changes in m^6^A Compared to Controls

**Figure 1:**
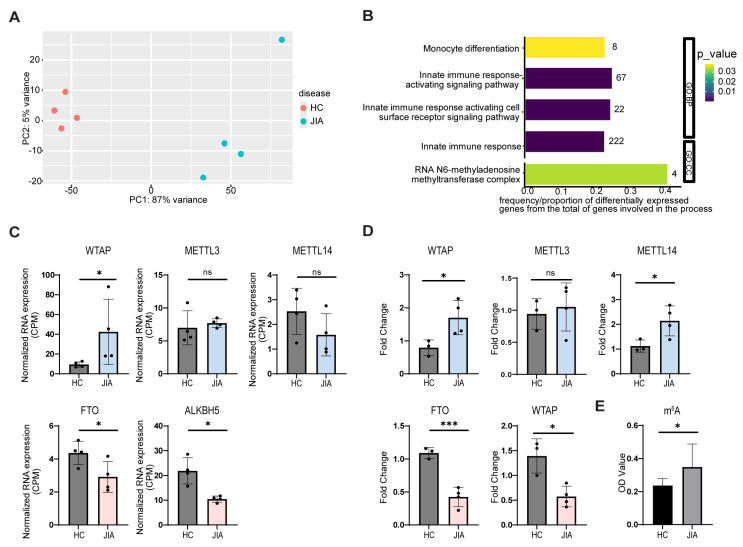
Changes in m^6^A-related genes and increased m^6^A methylation levels in JIA monocytes. RNA sequencing was performed on ex vivo CD14^+^ monocytes derived from oJIA patients or healthy controls (HC) to analyze changes in the expression of m^6^A-related genes. (**A**) Principal component analysis (PCA) of RNA-seq data. (**B**) Functional gene ontology enrichment analysis of differentially expressed genes for Gene Ontologies of Biological Process (GO:BP) and Cellular Component (GO:CC). The number of differentially expressed genes involved in the category are shown beside the bars. (**C**) Normalized RNA expression (CPM) for *WTAP*, *METTL3*, *METTL14*, *FTO*, and *ALKBH5* in oJIA and healthy control monocytes. (**D**) Relative mRNA expression of the same m^6^A regulators in CD14^+^ monocytes isolated from peripheral blood of healthy controls and synovial fluid of oJIA patients, measured by RT-qPCR and normalized to *B2M*. (**E**) Quantification of global m^6^A methylation levels in JIA and healthy monocytes using a colorimetric assay. *p* values were calculated using paired or unpaired T-tests as appropriate (* *p* < 0.05; *** *p* < 0.001; ns = not significant).

To explore the transcriptional landscape of monocytes in oligoarticular juvenile idiopathic arthritis (oJIA), we performed RNA sequencing on CD14^+^ monocytes isolated from the synovial fluid of oJIA patients and compared them to peripheral blood monocytes from healthy controls. Principal component analysis (PCA) revealed a clear separation between JIA and healthy monocyte transcriptomes, with the first principal component (PC1) accounting for the majority of variance (87%), indicating a distinct transcriptional profile in JIA synovial monocytes (Figure 1A).

To investigate the biological pathways contributing to this divergence, we performed Gene Ontology (GO) enrichment analysis on differentially expressed genes. This analysis highlighted significant enrichment of pathways related to monocyte differentiation, innate immune activation, and notably, the RNA N6-methyladenosine (m^6^A) methyltransferase complex (Figure 1B). Guided by the enrichment of m^6^A-related pathways, we specifically evaluated the expression of key m^6^A regulators. A significant downregulation of the expression of m^6^A erasers *FTO* and *ALKBH5* was observed (Figure 1C). Furthermore, *WTAP*, a key component of the methyltransferase complex, was significantly upregulated in JIA monocytes. These findings were validated in an independent cohort by RT-qPCR. Consistent with the RNA-seq results, *FTO* and *ALKBH5* were significantly reduced in JIA monocytes. *METTL3* expression remained unchanged (Figure 1D).

To determine whether these transcriptional changes translated into functional epitranscriptomic alterations, we quantified global m^6^A levels using a colorimetric assay. JIA monocytes exhibited significantly higher m^6^A methylation compared to healthy controls (Figure 1E), consistent with reduced demethylase expression. Together, these findings indicate that JIA synovial monocytes exhibit an altered balance of m^6^A writers and erasers, resulting in elevated m^6^A methylation and potentially contributing to their activated phenotype.

### 2.2. Synovial Fluid from JIA Patients Downregulates m^6^A Eraser Expression in Healthy Monocytes

**Figure 2:**
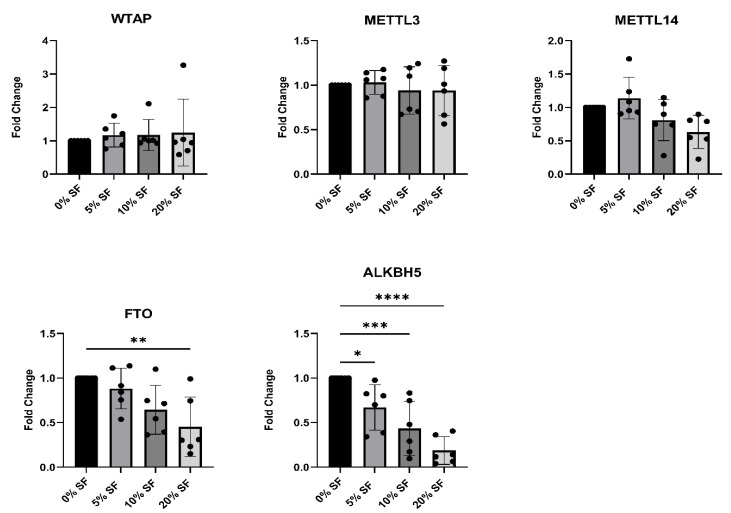
Synovial fluid from JIA patients modulates expression of m^6^A writers and erasers in healthy monocytes. CD14^+^ monocytes from peripheral blood of healthy controls were cultured with increasing concentrations (5%, 10%, 20%) of pooled synovial fluid (SF) derived from inflamed joints of oJIA patients for 3 h. mRNA expression of the m^6^A writers (*WTAP*, *METTL3*, *METTL14*) and erasers (*FTO*, *ALKBH5*) was measured by RT-qPCR and normalized to *B2M*. Statistical significance was assessed using one-way ANOVA (* *p* < 0.05; ** *p* < 0.01; *** *p* < 0.001; **** *p* < 0.0001).

To investigate whether the synovial microenvironment contributes to the altered expression of m^6^A regulators observed in JIA, we stimulated human CD14^+^ monocytes from healthy donors with increasing concentrations (5%, 10%, and 20%) of pooled synovial fluid (SF) derived from inflamed joints of oJIA patients. After three hours of stimulation the expression of the key m^6^A writers and erasers was assessed by RT-qPCR (Figure 2). The expression of the m^6^A erasers *FTO* and *ALKBH5* was significantly reduced by co-culture with synovial fluid in a dose-dependent manner, indicating that soluble factors present in JIA synovial fluid can suppress their expression. In contrast, expression levels of the writers *WTAP*, *METTL3*, and *METTL14* remained unchanged across all SF concentrations. These findings suggest that the inflamed synovial environment selectively downregulates m^6^A demethylases, potentially contributing to the elevated m^6^A methylation observed in JIA monocytes.

To further dissect which inflammatory mediators present in the synovial fluid might contribute to the reduced expression of *FTO* and *ALKBH5*, healthy donor monocytes were stimulated for 3 h with individual pro-inflammatory cytokines that have been reported to be present in the synovial fluid of JIA patients [31]. None of the tested cytokines alone were able to fully replicate the m^6^A regulator expression pattern observed in JIA synovial monocytes (Supplementary Figure S1), suggesting that a combination of inflammatory signals or other synovial fluid components drive the differences in expression of *FTO* and *ALKBH5* in the inflamed joint.

### 2.3. Genetic and Pharmacologic Inhibition of FTO Enhances TNFα Production in Monocytes

**Figure 3:**
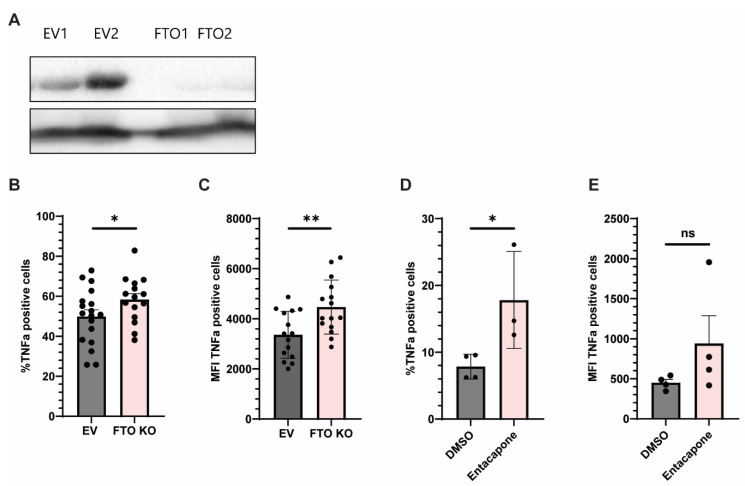
Genetic and pharmacologic inhibition of *FTO* enhances TNFα expression in monocyte-like cells. (**A**) Western blot showing protein expression of FTO in multiple independent CRISPR/Cas9 knockout clones (FTO1 and FTO2) and empty vector (EV1–EV3) controls. Histone H3 was used as a loading control. (**B**,**C**) Monomac6 cells with *FTO* knockout (*FTO* KO) or empty vector (EV) were stimulated with LPS (100 ng/mL, 3 h). Intracellular flow cytometry revealed a significant increase in both (**B**) the percentage of TNFα-positive cells and (**C**) the mean fluorescence intensity (MFI) of TNFα-positive cells in *FTO* KO clones.(**D**,**E**) Pharmacologic inhibition of FTO using entacapone (25 µM, 3 h) increased the percentage of TNFα-positive cells (**D**), with a non-significant trend in MFI (**E**), compared to DMSO-treated controls. Data represent pooled results from independent experiments. Error bars indicate mean ± SEM. Statistical significance was assessed using unpaired T-test (* *p* < 0.05; ** *p* < 0.01; ns = not significant).

Given the observed downregulation of m^6^A erasers *FTO* and *ALKBH5* in JIA synovial monocytes and their established role in inflammatory regulation, we aimed to assess whether the manipulation of *FTO* or *ALKBH5* expression could modulate monocyte activation, as measured by TNF production. We focused on TNF production as a readout because it is a rapid and robust hallmark of monocyte activation. Monocytes promptly secrete TNF upon stimulation, making it a sensitive and widely accepted marker for assessing activation status in this context. To this end, we performed CRISPR/Cas9-mediated knockout for both *FTO* and *ALKBH5* in Monomac6 cells, a human monocytic cell line. While *FTO* knockout was achieved, cells could not survive *ALKBH5* knockout. Western blot analysis confirmed successful *FTO* deletion across single-cell clones (FTO1 and FTO2), (Figure 3A). Upon stimulation with LPS (100 ng/mL, 3 h), *FTO* knockout (KO) clones exhibited increased TNFα expression compared to controls, both in the percentage of positive cells and in the mean fluorescence intensity (MFI) of TNFα-positive cells (Figure 3B,C), indicating enhanced cytokine production at the single-cell level.

To validate these findings, a pharmacologic inhibitor of FTO (entacapone) was used. Entacapone treatment significantly increased the percentage of TNFα-positive cells compared to DMSO controls (Figure 3D). Although MFI showed an upward trend, the difference did not reach statistical significance (Figure 3E).

These data collectively demonstrate that FTO can modulate TNFα production in monocyte-like cells, supporting a repressive role for FTO in the regulation of inflammatory cytokine expression. Together, these findings suggest that reduced expression of FTO, may contribute to enhanced TNFα production as observed in JIA monocytes at the site of inflammation.

## 3. Discussion

In this study, we assessed the role of m^6^A RNA methylation in Juvenile Idiopathic Arthritis (JIA) by analyzing monocytes isolated from the inflamed joints of oligoarticular JIA (oJIA) patients. Using RNA sequencing, we identified altered expression of several m^6^A machinery genes in JIA synovial monocytes, including a marked reduction in the expression of the m^6^A demethylase *FTO*. This transcriptional change was accompanied by increased global m^6^A levels on mRNA. Furthermore, stimulation of healthy monocytes with JIA synovial fluid led to reduced *FTO* expression, indicating that the synovial microenvironment directly influences m^6^A dynamics. Functional experiments confirmed that FTO plays a key regulatory role in monocyte activation, as both knockdown and knockout of *FTO* in healthy monocytes significantly increased TNF expression, highlighting its role in inflammatory cytokine regulation.

The dysregulation of m^6^A writers and erasers in JIA monocytes from inflamed joints underscores the role of m^6^A RNA methylation in the pathogenesis of this autoimmune disorder. These findings suggest that dysregulated m^6^A modifications may contribute to the aberrant inflammatory responses observed in JIA. Our findings align with emerging research implicating m^6^A RNA methylation in various autoimmune diseases and inflammatory disorders [16,19,24,26,28]. The reduced expression of *FTO*, and *ALKBH5* in JIA monocytes mirrors observations in other autoimmune disorders. For example, *FTO*, *ALKBH5* and *YTHDF2* have been described as risk factors for rheumatoid arthritis [22]. Since we observed decreased expression of both *FTO* and *ALKBH5* in JIA monocytes, there is a possibility that *FTO* and *ALKBH5* influence each other’s expression or function. Previous studies have indicated that erasers may act in a functional balance to fine-tune immune activation. For example, FTO has been linked to pro-inflammatory responses in macrophages [21,32], while ALKBH5 has been reported to modulate inflammatory pathways in other settings [33]. A recent review also indicates that erasers and writers may exert complementary or opposing effects in innate immune regulation [34]. This raises the possibility that similar dynamics could also exist in monocytes in JIA, although this remains to be investigated.

The role of FTO in modulating TNF expression and monocyte activation sheds light on a specific mechanistic pathway linking m^6^A RNA methylation to JIA pathogenesis. We propose that decreased *FTO* expression, and possibly also *ALKBH5*, may contribute to increased TNF production and monocyte hyperactivation, further promoting the chronic inflammation characteristic for JIA. Further research is necessary to investigate and identify the responsible m^6^A reader protein and molecular mechanism in JIA pathology.

The rapid downregulation in FTO expression in healthy monocytes exposed to JIA synovial fluid demonstrates the influence of the local microenvironment on m^6^A regulation. This finding suggests that factors within the synovial milieu actively drive gene expression changes and inflammation in JIA, potentially creating a feedback loop that sustains disease activity. However, the underlying mechanisms for *FTO* expression changes remain elusive. Both FTO and ALKBH5 are Fe^2+^-dependent enzymes [35,36], and altered iron homeostasis in the inflamed synovial environment could impair their catalytic activity. Inflammatory conditions are known to cause iron sequestration and imbalance, which may reduce the bioavailability of Fe^2+^ despite overall iron accumulation. Iron dysregulation has been reported in the synovial fluid of patients with inflammatory arthritis [37,38,39], suggesting that similar mechanisms may occur in JIA. Such dysregulation of iron could therefore contribute to impaired demethylase activity in JIA synovial monocytes. Assessing iron levels or iron-related markers in JIA synovial fluid will be required to address this possibility.

Future research could explore the potential therapeutic targeting of m^6^A modifiers, such as FTO, to mitigate inflammation in JIA. Our findings have significant clinical implications, as they highlight the potential of m^6^A RNA methylation as a therapeutic target in JIA. Modulating the m^6^A machinery, may offer a novel approach to dampen the inflammatory response and ameliorate disease progression in JIA patients [3].

## 4. Methods and Materials

### 4.1. Cell Culture

PBMCs and SFMCs were isolated from peripheral blood of healthy controls or synovial fluid of oligo articular JIA patients using Ficoll-Paque density gradient centrifugation (Cytiva, Uppsala, Sweden)**.** CD14^+^ cells were isolated from frozen PBMCs using magnetic activated cell sorting (MACS) with human CD14 microbeads (Miltenyi Biotec, Bergisch Gladbach, Germany). Monocytes and Monomac6 cells were cultured in RPMI 1640 medium supplemented with 10% FBS, 100 U/mL penicillin, 100 mg/mL streptomycin and 1% L-Glutamine (Gibco, Thermo Fisher Scientific, Waltham, MA, USA) at 37 °C in 5% CO_2_. Cells were activated with 100 ng/mL LPS (Sigma-Aldrich, St. Louis, MO, USA) for 2 (Monomac6 cells) or 3 h (Monocytes) in culture medium prior to RNA and protein analysis.

### 4.2. Culture with JIA Synovial Fluid

Synovial fluid (SF) was collected from inflamed knee joints of oJIA patients during clinical procedures and stored at −80 °C until use. Prior to stimulation, SF samples were thawed and pooled when indicated. CD14^+^ monocytes from healthy donors were seeded at a density of 1 × 10^6^ cells per well in 24-well plates and cultured under standard conditions. Cells were stimulated for 3 h with increasing concentrations of JIA synovial fluid (5%, 10%, or 20% *v*/*v*) in supplemented RPMI 1640 medium. After stimulation, cells were lysed in RLT buffer for RNA extraction and subsequent RT-qPCR analysis.

### 4.3. Next Generation RNA-Sequencing

RNA-sequencing raw count expression data used in this paper has been published in Boltjes et al., 2023 [40]. Counts belonging to the canonical isoform or, if not available, to the isoform with higher mean normalized read counts (baseMean) were used for the analysis. DESeq2 function from the R v4.4.1 Bioconductor package DESeq2 v1.44.0 [41] was used to normalize read counts, using the median of ratios method with the formula ~disease + sex, to correct disease-specific effect by sex-specific differences. DESeq was used to identify differentially expressed genes performing a Wald test: *p*-values were obtained using the results function and adjusted for multiple testing, using the False Discovery Rate (FDR) procedure. log2FC were shrunken using the default and recommended apeglm v1.26.1 [42] algorithm of the lfcShrink function. Genes with an adjusted *p* value < 0.05 and an at least double difference in expression between evaluated conditions (|shrunken log2FC| > 1) were considered as differentially expressed. Functional enrichment analysis was performed for differentially expressed genes using gprofiler2 v0.2.3 [43] correcting for multiple testing by FDR, and significance was considered with an adjusted *p*-value < 0.05.

### 4.4. Total m^6^A RNA Methylation Quantification

Total RNA is isolated from healthy control and JIA synovial derived monocytes using the RNeasy Mini Kit (Qiagen, Hilden, Germany). Total m^6^A levels were measured according to manufacturer’s protocol with the EpiQuik™ m^6^A RNA Methylation Quantification Kit (Epigentek, Farmingdale, NY, USA) and 200 ng input RNA [44,45].

### 4.5. Quantitative PCR (RT-qPCR)

Total RNA was extracted using the RNeasy Mini Kit (Qiagen) and reverse transcribed into complementary DNA (cDNA) using the iScript™ cDNA Synthesis Kit (Bio-Rad, Hercules, CA, USA), according to the manufacturer’s instructions. Quantitative PCR (RT-qPCR) was performed using SYBR Select Master Mix (Thermo Fisher Scientific) on a CFX96 Real-Time PCR Detection System (Bio-Rad). Reactions were run in technical duplicates or triplicates depending on the assay. Gene expression levels were normalized to *B2M* as a housekeeping gene, and relative expression was calculated using the ΔΔCt method. For cytokine stimulation experiments, *RPL13A* (Bio-Rad) was used as the reference gene for normalization. Primer sequences for *FTO*, *RPL13A*, *ALKBH5*, *WTAP*, *METTL3*, *METTL14*, *TNF*, and *B2M* are listed in Supplementary Table S1.

### 4.6. Cytokine Stimulation

To assess the effect of specific inflammatory mediators on the expression of m^6^A writers and erasers, CD14^+^ monocytes from healthy donors were stimulated for 3 h with individual cytokines or innate immune stimuli. The following final concentrations were used: lipopolysaccharide (LPS, 100 ng/mL; Sigma-Aldrich, L4391), phorbol 12-myristate 13-acetate (PMA, 25 ng/mL; Sigma-Aldrich, P8139), ionomycin (1 µg/mL; Sigma-Aldrich, I0634), S100A8/A9 complex (10 µg/mL; R&D Systems, Minneapolis, MN, USA), interleukin-1β (IL-1β, 10 ng/mL; PeproTech, Cranbury, NJ, USA), interleukin-6 (IL-6, 20 ng/mL; PeproTech), tumor necrosis factor-alpha (TNFα, 20 ng/mL; PeproTech), interleukin-18 (IL-18, 50 ng/mL; MBL International, Woburn, MA, USA), interferon-alpha2 (IFNα2, 1000 IU/mL; PeproTech), and interferon-gamma (IFNγ, 100 ng/mL; PeproTech). All stimulations were performed in RPMI 1640 medium supplemented with 10% FBS. After stimulation, cells were lysed for RNA extraction and subsequent gene expression analysis as described above. Each condition was tested using monocytes from at least three healthy donors.

### 4.7. Western-Blotting

Cells were lysed in Laemmli buffer (0.12 M Tris-HCl, pH 6.8, 4% SDS, 20% glycerol, 0.05 µg/µL bromophenol blue, 35 mM β-mercaptoethanol) and protein levels were normalized using the BCA protein assay kit (Thermo Scientific). SDS-PAGE separation was performed with a 10% gel and transferred to a polyvinylidene difluoride (PVDF) membrane (Merck, Darmstadt, Germany). Blots were incubated with rabbit anti human FTO (Abcam, Cambridge, UK; ab124892). Secondary anti-rabbit HRP (Agilent, Santa Clara, CA, USA; P026002-2) was used and blots were analyzed using chemiluminescence (Thermo Fisher Scientific). Detection was performed using enhanced chemiluminescence (Thermo Fisher Scientific). Histone H3 (Cell Signaling Technology, Danvers, MA, USA; 9715S) was used as a loading control.

### 4.8. CRISPR/Cas9 Knock-Out

Monomac6 cells were transduced with the lentiviral vector pSicoR-CRISPR-PuroR (RP-557) containing single-guide RNAs (sgRNAs) to induce *FTO* knock-out (CRISPR RNA sequence: 5′-GGCTGCTTATTTCGGGACC-3′). Virus was produced in HEK293T cells using polyethylenimine (PEI) MAX (Polysciences, Warrington, PA, USA) and second-generation lentiviral particles. Monomac6 cells were transduced using 5 µg/mL of polybrene (Santa Cruz Biotechnology, Dallas, TX, USA) and cells were single-cell-cultured and selected with 2 μg/mL puromycin (Merck) selection.

### 4.9. FTO Inhibition and Flow Cytometry

CD14^+^ monocytes from healthy donors were cultured in 96-well round-bottom plates at a density of 1 × 10^5^ cells per well and treated with 25 µM entacapone (Sigma-Aldrich) for 48 h. Control wells received matched concentrations of dimethyl sulfoxide (DMSO; Sigma-Aldrich, D2650). After pre-treatment, cells were stimulated with 100 ng/mL LPS (Sigma-Aldrich) for 3 h. GolgiStop™ (BD Biosciences, San Jose, CA, USA) was added 30 min after LPS stimulation to block cytokine secretion. Following stimulation, cells were fixed, permeabilized, and stained for intracellular TNFα using an APC-conjugated antibody (BioLegend, San Diego, CA, USA; clone MAb11, catalog #502912). Samples were acquired on a BD FACSCanto™ II flow cytometer and analyzed using FlowJo v10 (Tree Star Inc., Ashland, OR, USA).

## 5. Conclusions

In conclusion, our study provides evidence of dysregulated m^6^A RNA methylation in JIA monocytes and identifies FTO as a pivotal player in TNF regulation and monocyte activation. These findings broaden our understanding of the molecular mechanisms underlying JIA pathogenesis and offer potential therapeutic targets for the treatment of JIA and possibly other autoimmune diseases in the future.

## Data Availability

The original contributions presented in this study are included in the article/Supplementary Materials. Further inquiries can be directed to the corresponding author.

## Supplementary Materials

The following supporting information can be downloaded at: https://www.mdpi.com/article/10.3390/ijms26189248/s1.

## Abbreviations

The following abbreviations are used in this manuscript:

ALKBH5	AlkB Homolog 5
APC	Allophycocyanin
CRISPR	Clustered Regularly Interspaced Short Palindromic Repeats
DESeq2	Differential Expression Sequencing 2
DMSO	Dimethyl Sulfoxide
FBS	Fetal Bovine Serum
FTO	Fat Mass and Obesity-Associated Protein
GO	Gene Ontology
JIA	Juvenile Idiopathic Arthritis
LPS	Lipopolysaccharide
MACS	Magnetic-Activated Cell Sorting
METTL3	Methyltransferase-Like 3
METTL14	Methyltransferase-Like 14
MFI	Mean Fluorescence Intensity
m^6^A	N6-methyladenosine
PBMC	Peripheral Blood Mononuclear Cell
PCA	Principal Component Analysis
PEI	Polyethylenimine
RT-qPCR	Reverse Transcription Quantitative Polymerase Chain Reaction
RA	Rheumatoid Arthritis
RPMI	Roswell Park Memorial Institute Medium
RT	Reverse Transcription
SF	Synovial Fluid
SFMC	Synovial Fluid Mononuclear Cell
TNFα	Tumor Necrosis Factor Alpha
WTAP	Wilms Tumor 1-Associating Protein
