# Supplementary material for: Reduced Expression of m6A Demethylases FTO and ALKBH5 in Monocytes from the Site of Inflammation in Patients with Juvenile Idiopathic Arthritis
Source: Int J Mol Sci. 2025 Sep 22;26(18):9248. doi: 10.3390/ijms26189248 (PMC12471030; doi:10.3390/ijms26189248)
Supplement: Supplementary file 1 [file ijms-26-09248-s001.zip › ijms-3819754-supplementary.pdf]

Supplemental Table S1 – qPCR primers

Target gene	Forward (5' to 3')	Reverse (5' to 3')
METTL3	AGATGCGCAGGCTCAACATA	TACGGCCTGTCCGAATGATG
METTL14	AGCGTCAGTCTTCGTGGAGT	TCCAGGCGTAGCTTCACTTT
WTAP	TGCGACTAGCAACCAAGGAA	GTTGATCGCTGGGTCTACCA
FTO	ATGCTTGATGATCTCAATGCCACC	ACTGCAGGCTCAAAGGATTTC
ALKBH5	ACTGTGCTCAGTGGATATGCT	AGCTGCTCAGGGACTTTGTT
YTHDF1	ACACCCAGAGAACAAAAGGACA	CCGTAGGTGGTGAGGTATGG
β2M	GGCATCTTCAAACCTCCATG	ATGAGTATGCCTGGCCGTGTGA
TNF	CCCCAGGGACCTCTCTCTAA	TGAGGTACAGGCCCTCTGAT
RPL13A	CCTGGAGGAGAAGAGGAAAGAGA	TTGAGGACCTCTGTGTATTTGTCAA

Supplemental Figure S1.

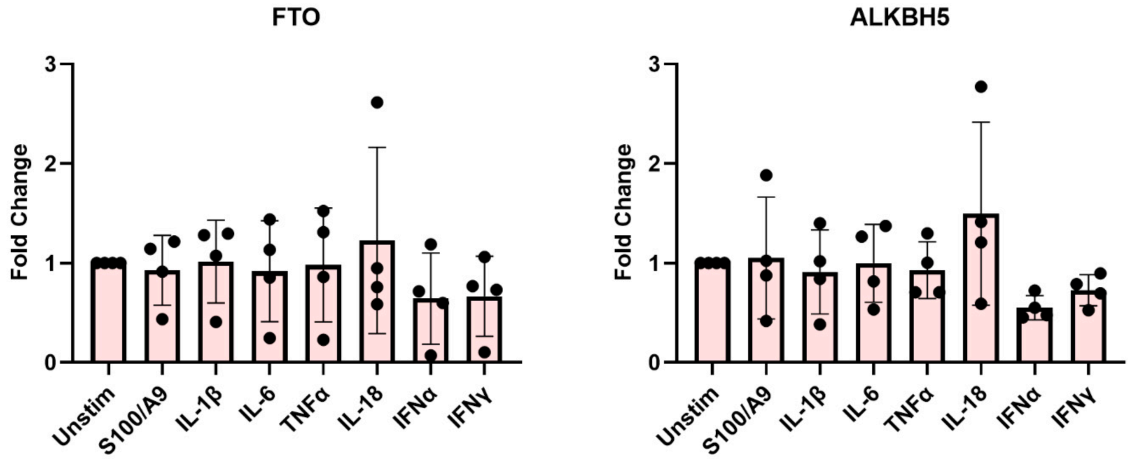

Supplemental Figure S1. Cytokine stimulation of healthy monocytes does not fully recapitulate m⁶A regulator expression changes observed in synovial monocytes. Primary CD14⁺ monocytes from healthy donors were stimulated for 3 hours with various inflammatory cytokines. Gene expression of selected m⁶A regulators FTO and ALKBH5 was measured by RT-qPCR and normalized to RPL13A expression. Bars represent mean ± SEM from independent donors. . Statistical significance was assessed using one-way ANOVA ($p < 0.05$; $p < 0.01$; ns = not significant.
